# Multidomain Narrowband Interference Intelligent Suppression Method Based on Cognitive Radio and MIMO in UAV Data Link

**DOI:** 10.1155/2022/6207937

**Published:** 2022-05-05

**Authors:** Xianquan Luo, Ling You, Junwei Lv, Chen Zhong

**Affiliations:** ^1^College of Artificial Intelligence, Yango University, Fuzhou, Fujian 350015, China; ^2^Jiangsu College of Engineering and Technology, Nantong, Jiangsu 226000, China

## Abstract

In this paper, a kind of antinarrowband interference intelligent method based on cognitive MIMO (multiple-input multiple-output) airspace, time domain, frequency domain, and code domain is proposed. This method combines perceptual technology, spread spectrum code, MIMO space-time coding, and so on. Through coordinating space, time, frequency, and code element diversity, the narrowband interference in the UAV (unmanned aerial vehicle) monitoring and control link is effectively countered. The input and output model of MIMO monitoring and control link of UAV with natural interference are described. Then, the realization principle of the multidomain antinarrowband interference method based on CR-MIMO is presented, and the corresponding models of receiving and transmitting are also given. Finally, the anti-interference performance of the proposed method is analyzed theoretically and validated through simulation experiments, and the effectiveness of the proposed method is verified.

## 1. Introduction

In the wireless MIMO communication system, orthogonal space-time block coding is a typical application example of MIMO diversity gain, which has the advantage of low complexity. It can improve the reliability of data link system through using the MIMO orthogonal space-time block coding. But in a variety of hostile interference cases, especially when the interference power is very strong, as the MIMO orthogonal space-time block coding cannot be effective against hostile interference, its performance will be affected accordingly.

In this case, it is necessary to adopt corresponding processing method to suppress interference. Because MIMO technology in the application of the UAV data link is relatively late, the UAV needs to deal with the hostility of MIMO channel interference; on the one hand, the public research methods are relatively few. However, it is difficult to use one technique or method to suppress all interference.

In the paper, the multidomain narrowband interference suppression method based on cognitive radio is proposed through the antijamming simulation experiments of typical narrowband noise, the number of continuous waves, frequency sweep and broadband comb, broadband noise, and interference suppression of the CR-MIMO multidomain. The results show that this technology can make full use of interference spectrum and realize a class effective using of the spectrum characteristics of interference and the interference suppression ability is good.

In the paper, it firstly studies the corresponding suppression methods for the typical narrowband interference under hostile interference. In the second part, the newly related work is discussed and analysed; the proposed method is also introduced. In the third part, UAV data link based on MIMO diversity is analysed deeply and its characteristics are introduced. In the fourth part, multidomain narrowband interference suppression method is also introduced and presented.

In the fifth part, system error rate is obtained through related experiments. And some useful conclusions are drawn and given out.

## 2. Related Work Discussion and Analysis

Narrowband interference mainly includes single tone, multitone, continuous wave interference, sweep frequency interference, and noise interference [1]. Current interference signals are weakened mainly through the spread spectrum processing section, but as the interference is commonly overlapped with the signal spectrum, the method cannot completely eliminate the interference effect of the signal, especially when the narrowband interference power is large, and spread spectrum gain needed is also very big, and the processing cost will be increased accordingly. And the current research of interference avoidance is an effective method to deal with narrowband interference.

Its basic idea is under the condition of known hostile interference frequency range, through the method of avoiding to make useful signal and interference signal orthogonal in frequency domain. Among them, frequency evading mainly adopts cognitive radio (CR) technology [2], which can adjust the parameters of the surrounding radio spectrum to realize anti-interference in the frequency domain. Transform domain communication system is a typical application of this technology, which can make real-time perception in a certain communication bandwidth among the frequency spectrum of the electromagnetic environment, it also can dynamically generate modulation function, which does not contain the interference spectrum, and it is used to transfer information so as to realize active interference avoidance.

Some researchers have put forward to transform domain communication combined with MIMO V-BLAST spectrum sharing, which can avoid single tone, sound, and narrowband interference, but its coding diversity gain is small, and it cannot be effective against natural interference. Sundaravadivu et al. [3] proposed sensing combined with MIMO precoding to achieve beam informs, which can avoid MIMO system work in the other direction from the airspace of communication interference, and the method in the face of multiple interference, the interference is inevitable, it needs to take further anti-interference measures.

In this paper, a joint narrow band interference method based on cognitive MIMO in space, time, frequency, and code domains is studied. This method combined with perception technology, MIMO space-time coding, and spread spectrum code through coordination of space, time, frequency, and element diversity effectively against the narrow-band interference of the UAV data-link.

The input and output model of MIMO data link with natural interference and enemy interference are introduced, and the realization principle of forward space time packet coding and decoding is discussed in detail. Then, the realization principle of MIMO multidomain antinarrowband interference method and the corresponding model of receiving and starting are also presented. Finally, the anti-interference performance of the proposed method is analyzed theoretically and validated by simulation experiments.

In the paper [4], frequency comb calibrated frequency-sweeping interferometry for absolute group refractive index measurement of air is presented. Determination analysis of minimum requirements of the data link communication system for unmanned aerial vehicles (UAVs) is also given out.

Mitigating jamming attacks in wireless broadcast systems is introduced in the paper [5]; in [6–9], antijamming performance simulation and analysis of tactical data link communication system and security of classic PN-spreading codes for hybrid DS/FH spread-spectrum systems are discussed.

In the papers [4, 10–18], resistance to interference for OFDM-based cognitive radio systems and expectation propagation for near-optimum detection of MIMO-OFDM signals are analyzed, and the OFDM-based cognitive radio is an effective method for the antijamming. Aeronautical channel modeling and simulation are researched deeply, and the analysis and simulation of typical mode of jamming on data link communication system is proposed.

In the papers [19–24], robust minimum dispersion distortionless response beamforming against fast-moving interferences in the aircraft application are introduced and electromagnetic interference suppression method of motor assembly for aircraft application is also analyzed deeply and show that MIMO-OFDM signals processing methods are the trend in the antijamming system.

In the papers [25–29], many methods for interference suppression and signal acquisition in the communication systems are introduced and analyzed, and the cognitive radio and MIMO in UAV communication data link will be the effectiveness method and the trend in the near future.

## 3. UAV Data Link Based on MIMO Diversity

### 3.1. The Principle of Encoding and Decoding of MIMO under Natural Disturbance

Assume that the number of antennas in the UAV MIMO remote control link is *m*_*T*_, the number of receiving antennas is *m*_*R*_, and the channel is flat fading type. In a moment of *T*, the input and output model of the system is as follows.

(1) $$\begin{array}{c} R=\rho HX+N. \end{array}$$

Among them, $\rho =\sqrt{E_{0}/m_{T}}$, *E*_0_ is the total output power; *R* is the *m*_*R*_ × *T* dimensional reception signal; *H* is the *m*_*R*_ × *m*_*T*_ dimensional MIMO channel matrix; *N* denotes the *m*_*R*_ × *T* gaussian white noise matrix whose elements are distributed by zero-mean circularly symmetric complex gaussian (ZMCSCG) distribution. The variance is *n*_0_. Orthogonal STBC encoding algorithm is used to send the sequence *X* for *m*_*T*_ × *T* dimension.

**Figure 1:**
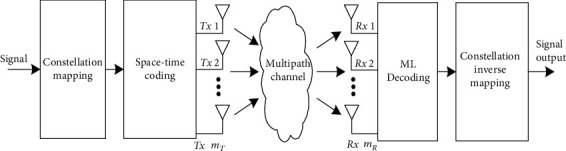
Schematic diagram of MIMO orthogonal space-time block coding.

The orthogonal STBC coding algorithm is used to transmit the sequence. Through this algorithm, it makes the coding matrix column in different orthogonal transmitting signals to maintain orthogonality, it adopts two-dimensional joint diversity in space and time to achieve full diversity gain, the receiver simply through maximum likelihood algorithm to decode, and the diversity gain and implementation complexity are both considered; its implementation principle is as shown in Figure 1 [30].

The decoding principle is the process can be realized by calculating the estimated value of the sending code element and the maximum value of all points in the constellation to get estimate way of element discrimination. Send code estimation is based on the space-time receiving matrix combined with channel matrix trace and makes simple merge for local diversity receiving antenna vector and vector channel.

### 3.2. Principle of Space-Time Decoding of Hostile Interference

**Figure 2:**
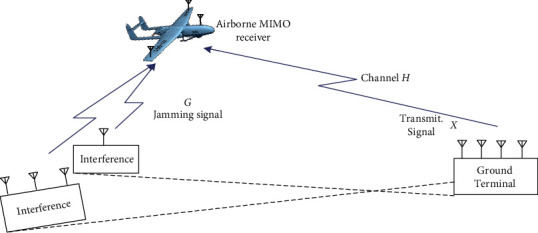
Sketch map of UAV-MIMO telecontrol link of enmity jamming.

When there is hostile interference to UAV-MIMO data link, as the uncertainty of the interference, so the interference may have multiple directions. It also may have multiple antennas on the same direction; finally, the interference of airborne receiver may have multiple paths as shown in Figure 2. In order to facilitate subsequent analysis, the possible hostile interference in the data link is considered as a whole at the receiving part.

In the case of hostile interference, the receiving antenna matrix is

(2) $$\begin{array}{c} R=\rho HX+G+N. \end{array}$$

Among them, *G* is *m*_*R*_ × *T* the dimension interference signal; its elements satisfy the independent same distribution. If the receiver has known channel information, the MLD method should be as below.

(3) $$\begin{array}{c} {\tilde{x}}_{ML}=\arg\underset{x_{p}\in S}{\min}{\left\|R-G-\rho HX\right\|}_{F}^{2}\\ =\arg\underset{x_{p}\in S}{\min}tr{\left(R-G-\rho HX\right)}^{H}\left(R-G-\rho HX\right). \end{array}$$

It can be seen that the MLD method needs accurate interference information to eliminate its influence on the MLD symbol decision. However, due to the complexity and uncertainty of interference, it is difficult for the receiver to obtain accurate interference information. Therefore, it is difficult to realize the effective application of MLD method in UAV MIMO datal link under hostile jamming conditions. Therefore, it is necessary to study the corresponding antijamming measures to suppress or eliminate the influence of jamming on MLD.

## 4. Multidomain Narrowband Interference Suppression Method

### 4.1. Principle of Multidomain Anti-Interference Based on MIMO

**Figure 3:**
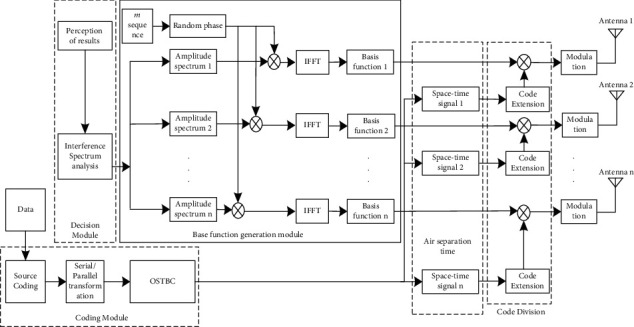
Transmitter diagram of multidomain antijamming based on CR-MIMO.

In order to make the suppression of narrowband interference in the UAV data link, it adopts MIMO multidomain anti-interference methods; its principle is as shown in Figure 3. It mainly involves decision module, basis function generating module, coding module, and spread spectrum. Firstly, the environmental perception parameters are obtained, and the spectrum is analysed. The perceptual results are obtained in the decision-making module. In the coding module, the information encoded by the source code is serialized and transformed according to the number of antennas, and then the parallel data is spatially coded to obtain the spatial and time diversity signals. When the space-time signal is realized by different sequence spread spectra, it is multiplied by the base function that is orthogonal to the interference generated by the base function generation module. Finally, the information is modulated to the corresponding transmitting antenna.

**Figure 4:**
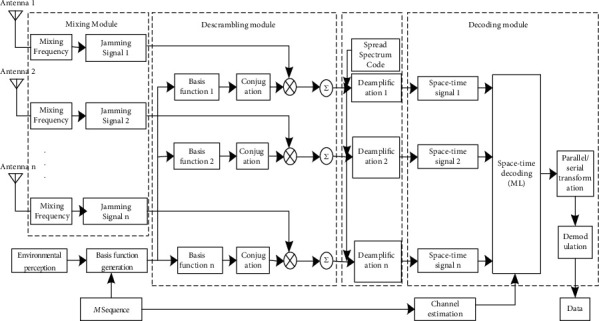
Receiver diagram of multidomain antijamming based on CR-MIMO.

The airborne receiving principle of the measurement and control link is shown in Figure 4, which mainly includes the sensing module, channel estimation, mixing module, scrambling module, and decoding module. Spectrum perception airborne antenna is first conducted to obtain the interference spectrum; this information needs to link data shared with the ground control station transmitting terminal; it adopts the same as the sender of m sequence and generates the basis function of the same orthogonal characteristics and interference. Then, the receiving signal is mixed frequency by demodulation module, and the information with interference and noise is obtained. The disassembly module can multiply the demodulation signal and the conjugate base function and obtain the undisturbed receiving signal, and the decoder module will further expand the decoding signal of the emission so as to reduce the influence of the interference. Finally, the decoding module is combined with the channel estimation to decode the space-time signal, and the transmitting data is obtained through the conversion and demodulation.

### 4.2. Multidomain Anti-Interference Mathematical Model Based on MIMO

#### 4.2.1. Transmitter Mathematical Model Scale Figures

According to the principle of UAV CR-MIMO multidomain antijamming, airborne antenna radio perception is the premise of realizing intelligent anti-interference. The two methods, ambient energy method based on FFT [30] and the power spectrum estimation method of combining the spectrum analysis [31], have been the interference frequency range “or” operation, and then it gets more accurate interference frequency range *f*_*G*_1__ ~ *f*_*G*_2__. The number of antennas is defined as Num. The frequency of transmitting frequency of the *i*th antenna is *f*_*i*_ the bandwidth *B*_*i*_, and the antenna and its RF system work interference tolerance *δ*.

According to the frequency range of the interference, it can be determined by formula (4) whether (Yes or No) there is a range of frequencies available.

(4) $$\begin{array}{c} \left\{\begin{array}{c} \delta *\left(f_{i}\pm \frac{B_{i}}{2}\right)\in f_{G_{1}}∼f_{G_{2}}\;\text{No,}\\ \delta *\left(f_{i}\pm \frac{B_{i}}{2}\right)\notin f_{G_{1}}∼f_{G_{2}}\;\text{Yes}. \end{array}\right. \end{array}$$

The amplitude spectrum of the *i*th antenna *A*_*k*_^*i*^ (*k*=1,2,…, *N*) is obtained according to the frequency range of the interference. In a sample point *N*, if the spectrum of a point is disturbed, the value of the point is set to 0; instead set it as 1. In order to compensate for the perceived missing frequency, the range of the perceived interference frequency can be appropriately broadened, namely, *f*_G_1__ − Δ_1_ ~ *f*_*G*_2__+Δ_2_; the amplitude spectrum of the *i*th antenna composed of 0 and 1 can be obtained as **A**^**i**^(*ω*)={*A*_1_^*i*^, *A*_2_^*i*^,…, *A*_*N*_^*i*^}.

The random phase encoding of amplitude spectrum makes it have the waveform with similar noise and also improves the characteristics of LPI (Low Probability of Intercept) in UAV communication. It adopts linear feedback shift register here of m sequence delay phase map generating (different transmitting antenna can generate different *m* sequence, and each gets a different random phase) according to the MPSK modulation to generate random phase (*e*^*j*2*πm*_*k*_/*M*^*M*=2^*r*^, 0 ≤ *m*_*k*_ ≤ 2^*r*^ − 1).

According to the transmitting power requirement, the sending code element is adjusted for energy, and each code element is used for the same power. The emission energy of the *i*th antenna (for each code element energy, the number of “1” in the amplitude spectrum vector). Finally, the discrete basis function of the frequency domain is obtained, and the inverse transformation is carried out to obtain the basis function of discrete time domain.

(5) $$\begin{array}{c} b_{i}\left(n\right)=\frac{1}{N}\sum_{k=1}^{N}\sqrt{\frac{\varepsilon N}{N_{A^{i}}}A_{k}^{i}e^{j^{j2\pi m_{k}/M}}e^{j^{j2\pi n_{k}/n}}}\;i=1,2,\ldots ,\text{Num}. \end{array}$$

Digital modulation of sending data, serial sequence of modulation is *x*_1_, *x*_2_, *x*_3_…. The parallel sequence (*x*_1_, *x*_2_,…,*x*_Num_)^*T*^ is generated according to the number of antennas. Then, the parallel sequence is space-time coded, and the encoding method is like equation (2). The transmitting signal of antenna I at *t* time is *x*_*i*_^*t*^ at the time of *t*, and the signal is amplified by the sequence length of *L* the periodic sequence ${\tilde{m}}^{i}$ (all the transmitting antennas here use the same spread spectrum sequence) (*x*_1_, *x*_2_,…,*x*_Num_)^*T*^. Finally, the base function is modulated into the corresponding emission sequence, and the transmitting sequence of carrier modulation by the *i*th antenna is as below:

(6) $$\begin{array}{c} x_{i}^{t}{\tilde{m}}^{i}\left(l\right)b_{i}\left(n\right)\cos\left(2\pi f_{i}t\right). \end{array}$$

#### 4.2.2. Mathematical Model of Receiving Part

Receiving signal of the antenna received by the aircraft is transmitted through the MIMO channel transmission of the UAV.

(7) $$\begin{array}{c} r^{j}\left(nl\right)=\sum_{i}^{\text{Num}}h_{ji}\times x_{i}^{t}{\tilde{m}}^{i}\left(l\right)b_{i}\left(n\right)\cos\left(2\pi f_{i}t\right)+g^{j}\left(nl\right)+n^{j}\left(nl\right), \end{array}$$

where *g*^*j*^(*n*) is the interference signal of the first person *j* antenna; 1 ≤ *l* ≤ *L*.Through the local carrier mixing frequency, the low pass filter is used to eliminate the high-frequency items, and the receiving signals ${\tilde{r}}^{j}\left(nl\right)$ with interference and noise are obtained as follows:

(8) $$\begin{array}{c} {\tilde{r}}^{j}\left(nl\right)=\frac{1}{2}\sum_{i}^{\text{Num}}h_{ji}\times x_{i}^{t}{\tilde{m}}^{i}\left(l\right)\times b_{i}\left(n\right)+g^{j}\left(nl\right)\text{cos}\left(2\pi f_{i}t\right)+n^{j}\left(nl\right)\text{cos}\left(2\pi f_{i}t\right). \end{array}$$

Adopt the same as the transmitter of spread spectrum sequence, according to the environmental awareness of the spectrum, to generate the same as the transmitter on the ground of basis functions (assuming the start timing), to take conjugate base function *b*_*i*_^*∗*^(*n*), and multiply with ${\tilde{r}}^{j}\left(nl\right)$; the sum can be obtained.

(9) $$\begin{array}{c} S^{j}\left(l\right)=\sum_{n=1}^{N}{\tilde{r}}^{j}\left(nl\right)b^{*}\left(n\right)\\ =\sum_{n=1}^{N}\left[\frac{\varepsilon }{2NN_{A^{i}}}\sum_{k=1}^{N}{\left(A_{k}^{i}\right)}^{2}\times \sum_{i}^{\text{Num}}h_{ji}\times x_{i}^{t}{\tilde{m}}^{i}\left(l\right)\right]+\sum_{n=1}^{N}g^{j}\left(nl\right)\text{cos}\left(2\pi f_{i}t\right)b^{*}\left(n\right)+\sum_{n=1}^{N}n^{j}\left(nl\right)\text{cos}\left(2\pi f_{i}t\right)b^{*}\left(n\right)\\ =\frac{\varepsilon }{2}\sum_{i}^{\text{Num}}h_{ji}\times x_{i}^{t}{\tilde{m}}^{i}\left(l\right)+\;\sum_{n=1}^{N}g^{j}\left(nl\right)\text{cos}\left(2\pi f_{i}t\right)b^{*}\left(n\right)+\sum_{n=1}^{N}n^{j}\left(nl\right)\text{cos}\left(2\pi f_{i}t\right)b^{*}\left(n\right). \end{array}$$

In the same way, the same spread spectrum sequence and equation (10) are used to solve the expansion. According to the autocorrelation property of the sequence, there is

(10) $$\begin{array}{c} R^{j}=\frac{1}{L}\sum_{l=1}^{L}S^{j}\left(l\right){\tilde{m}}^{i}\left(l\right)\\ =\frac{\varepsilon }{2}\sum_{i}^{\text{Num}}h_{ji}\times x_{i}^{t}+\frac{1}{L}\sum_{l=1}^{L}\sum_{n=1}^{N}g^{j}\left(nl\right)\cos\left(2\pi f_{i}t\right)b^{*}\left(n\right){\tilde{m}}^{i}\left(l\right)+\frac{1}{L}\sum_{l=1}^{L}\sum_{n=1}^{N}n^{j}\left(nl\right)\cos\left(2\pi f_{i}t\right)b^{*}\left(n\right){\tilde{m}}^{i}\left(l\right). \end{array}$$

*R* ^*j*^ is the space-time decoding result. And it can be simplified to

(11) $$\begin{array}{c} R^{j}=\sum_{i}^{\text{Num}}h_{ji}\times x_{i}^{t}+{\tilde{g}}^{j}+{\tilde{n}}^{j}. \end{array}$$

Among them,

(12) $$\begin{array}{c} {\tilde{g}}^{j}=\frac{2}{\varepsilon L}\sum_{l=1}^{L}\sum_{n=1}^{N}g\left(nl\right)\cos\left(2\pi f_{i}t\right)b^{*}\left(n\right){\tilde{m}}^{i}\left(l\right), \end{array}$$

(13) $$\begin{array}{c} {\tilde{n}}^{j}=\frac{2}{\varepsilon L}\sum_{l=1}^{L}\sum_{n=1}^{N}n^{j}\left(nl\right)\cos\left(2\pi f_{i}t\right)b^{*}\left(n\right){\tilde{m}}^{i}\left(l\right). \end{array}$$

If interference analysis can be accurate perception UAV by human disturbance, depending on the sending and receiving the basis function, the generating principle of human disturbance signals and orthogonal basis function amplitude, so type (12) is 0. Finally, equation (13) can obtain the null signal of the *j*-root antenna.

(14) $$\begin{array}{c} R^{j}=\sum_{i}^{\text{Num}}h_{ji}\times x_{i}^{t}+{\tilde{n}}^{j}\left(n\right). \end{array}$$

Directly to ${{\tilde{x}}_{i}/\Phi }_{1}$ ML decoding can eliminate the signal less susceptible to multipath channel; among them, Φ_1_ can be got through the method of least square channel estimation method [32–35]; the method for training sequences directly in the firing sequence modulation demodulation can avoid interference. Finally, the parallel data is transformed into serial data and demodulated to obtain the sequence sent.

### 4.3. Theoretical Analysis of Multidomain Anti-Interference Performance Based on CR-MIMO

According to the mathematical model of the receiver, the mean power of the valid information in the combined output is

(15) $$\begin{array}{c} E_{s}=\Phi_{1}^{2}E\left[{\left|x_{i}\right|}^{2}\right]=\varepsilon \Phi_{1}^{2}. \end{array}$$

The mean power of noise in the combined output is

(16) $$\begin{array}{c} E_{N}=E\left\{{\left[N_{i}\right]}^{2}\right\}\\ =E\left\{n^{j}\left(n\right){\left[n^{j}\left(n\right)\right]}^{*}\right\}\\ =4n_{0}\Phi . \end{array}$$

The dry ratio of the received letter is

(17) $$\begin{array}{c} \text{SJR}=\frac{E_{s}}{E_{G}+E_{N}}\\ =\frac{\varepsilon \Phi_{1}^{2}}{E_{G}+4n_{0}\Phi }. \end{array}$$

If the airborne antenna can detect the interference spectrum accurately, the reception signal is improved after the interference module is disturbed. If perception of residual spectrum, *E*_*G*_=0, the type (17) of the interference signals and incomplete orthogonal basis function amplitude, but after expansion code solution, interfering power *g*(*n*) can be suppressed; it also improves the receiving letters than done and gives play to the role of the expansion code anti-interference. Through the final simplification of the combined ML decoding, the influence of the noise on the signal can be suppressed so as to obtain the maximum acceptable dry ratio.

## 5. System Error Rate

Interference, noise, and channel estimation are the main factors that influence the system error performance in the multidomain antijamming method based on CR-MIMO. The system has effectively suppressed the interference, and the influence of the channel on the system's error performance is analyzed.

Combining with the characteristics of UAV data-link communication, its channel mainly includes the direct and scattering components, and it can therefore assume that the UAV rice fading channel could be as averages consisted of a real part and imaginary part and variance as complex Gaussian variables, which is subjected to degrees of freedom for the distribution center and its probability density function (PDF).

(18) $$\begin{array}{c} f\left(\varphi \right)=\frac{1}{2\sigma^{2}}{\left(\frac{\varphi }{2{\text{Num}}^{2}\sigma^{2}K}\right)}^{{\text{Num}}^{2}-1/2}\times e^{-KNum^{2}-\varphi /2\sigma^{2}}I_{{\text{Num}}^{2}-1}\left(\frac{\sqrt{2K{\text{Num}}^{2}\varphi }}{\sigma }\right). \end{array}$$

Among them, let parameter $K={\bar{\varepsilon }}_{I}^{2}+{\bar{\varepsilon }}_{Q}^{2}/2\sigma^{2}$, *I*_*α*_(*x*) is the Bessel function: *I*_*α*_(*x*)=∑_*m*=0_^*∞*^(*x*/2)^*α*+2*m*^/*m*!Γ(*α*+*m*+1).

(19) $$\begin{array}{c} f\left(\varphi \right)=\sum_{m=0}^{\infty }\frac{{\left(K{\text{Num}}^{2}\right)}^{m}e^{-K{\text{Num}}^{2}}e^{-\varphi /2\sigma^{2}}\varphi^{{\text{Num}}^{2}+m-1}}{\Gamma \left(m+1\right)\Gamma \left({\text{Num}}^{2}+m\right){\left(2\sigma^{2}\right)}^{{\text{Num}}^{2}+m}}. \end{array}$$

In ML decoding, the instantaneous symbol error probability is

(20) $$\begin{array}{c} P\left(X,\overset{⌢}{X}|H\right)=Q\left(\sqrt{\frac{E_{S}}{2E_{N}}d_{E}^{2}\left(X,\overset{⌢}{X}\right)}\right). \end{array}$$

As for $d_{E}^{2}\left(X,\overset{⌢}{X}\right)$ space-time code word matrix least-squares Euclidean distance, because this system adopts simplified ML decoding at the receiving part, namely, decoding of each code word, not for each element matrix, so the minimum Euclidean distance square turns into a single code word. If BPSK signal is adopted, the symbol error probability is [36]

(21) $$\begin{array}{c} P_{e}=Q\left(\sqrt{\frac{2E_{S}}{E_{N}}}\right)\\ =Q\left(\sqrt{\frac{2\varepsilon \Phi }{n_{0}}}\right). \end{array}$$

As for the expectation of false symbol rate, the error rate of the CR-MIMO multidomain anti-interference system is

(22) $$\begin{array}{c} P_{e}=\int_{0}^{+\infty }Q\left(\sqrt{\frac{\varepsilon^{3}\Phi }{2n_{0}}}\right)f\left(\varphi \right)\text{d}\varphi . \end{array}$$

Let $F\left(N\right)=\int_{0}^{+\infty }Q\left(\sqrt{ax}\right)x^{N-1}{\text{e}}^{-x/b}\text{d}x$.

(23) $$\begin{array}{c} F\left(N\right)=F\left(N\right)=\int_{0}^{+\infty }Q\left(\sqrt{ax}\right)x^{N-1}{\text{e}}^{-x/b}\text{d}x\\ =\int_{0}^{+\infty }be^{-x/b}\left(Q\left(\sqrt{ax}\right)x^{N-1}\right){}^{\prime }\text{d}x\\ =b\left(N-1\right)\int_{0}^{+\infty }Q\left(\sqrt{ax}\right)x^{N-2}{\text{e}}^{-x/b}\text{d}x-b\sqrt{\frac{a}{8\pi }}\int_{0}^{+\infty }x^{N-3/2}e^{-\left(2+ab/2b\right)x}\text{d}x\\ =b\left(N-1\right)f\left(N-1\right)-b{\left(\frac{1}{\lambda }\right)}^{N-1/2}\sqrt{\frac{a}{8\pi }}\int_{0}^{+\infty }{\left(\lambda x\right)}^{N-1/2-1}e^{-\lambda x}\text{d}\left(\lambda x\right). \end{array}$$

Among them, *λ*=(2+*ab*)/2*b*; the gamma (Γ) function is defined as

(24) $$\begin{array}{c} \Gamma \left(n\right)=\int_{0}^{+\infty }t^{n-1}e^{-t}\text{d}t. \end{array}$$

Therefore,

(25) $$\begin{array}{c} F\left(N\right)=b\left(N-1\right)f\left(N-1\right)-b{\left(\frac{2b}{2+ab}\right)}^{N-1/2}\Gamma \left(N-\frac{1}{2}\right)\\ =b^{N}f\left(1\right)-b^{N+1/2}\sqrt{\frac{a}{8\pi }}\sum_{l=0}^{N-1}{\left(\frac{2}{2+ab}\right)}^{N-l-1/2}\frac{\Gamma \left(N\right)\Gamma \left(N-l-1/2\right)}{\Gamma \left(N-l\right)},\\ F\left(1\right)=\frac{1}{2}\left(b-\sqrt{\frac{ab^{3}}{2+ab}}\right)\\ f\left(N\right)=\frac{1}{2}b^{N}\Gamma \left(N\right)\left[b-\sqrt{\frac{ab^{3}}{2+ab}}\right.-\left.\sqrt{\frac{ab}{2\pi }}\sum_{l=0}^{N-1}\frac{2^{N-l-1/2}\Gamma \left(N-l-1/2\right)}{{\left(2+ab\right)}^{N-l-1/2}\Gamma \left(N-l\right)}\right]. \end{array}$$

The system error rate can be obtained in theory asfollows.

(26) $$\begin{array}{c} P_{e}=\int_{0}^{+\infty }Q\left(\sqrt{2\varepsilon \varphi /n_{0}}\right)f\left(\varphi \right)\text{d}\varphi \\ =\sum_{m=0}^{\infty }\frac{{\left(K{\text{Num}}^{2}\right)}^{m}e^{-K{\text{Num}}^{2}}}{\Gamma \left(m+1\right)}\times \left[\sigma^{2}-\sqrt{\frac{2\varepsilon \sigma^{6}}{n_{0}+2\varepsilon \sigma^{2}}}-\sqrt{\frac{\varepsilon \sigma^{2}}{2\pi n_{0}}}\sum_{l=0}^{{\text{Num}}^{2}+m-1}\frac{n_{0}^{{\text{Num}}^{2}+m-l-1/2}\Gamma \left(Num^{2}+m-l-1/2\right)}{\Gamma \left({\text{Num}}^{2}+m-l\right){\left(n_{0}+2\varepsilon \sigma^{2}\right)}^{{\text{Num}}^{2}+m-l-1/2}}\right]. \end{array}$$

## 6. Simulation Experiments

**Figure 5:**
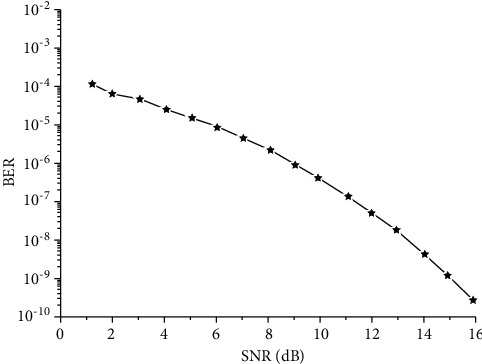
Relationship of SNR and theory bit error rate.

Make definition that fading channel transmission channel is rice channel, rice parameters *K*=5 (direct path), variance *σ*^2^=1, if the number of antennas is Num=4, SNR (signal-to-noise ratio) 1∼16 dB, element energy is *ε*=1, simulate the relationship between SNR and BER, set *m* = 100, as shown in Figure 5. With the increase of SNR, the error rate is gradually reduced.

Make the assumption that it randomly generates 12000 sent data at the sending part, the Monte Carlo number is 100, the period of *m* sequence is 512, and the sensing module can obtain the interference range accurately. In the typical application of cognitive anti-interference TDCS communication [37, 38], TDCS-MIMO communication and the DSSS anti-interference method mainly adopted by UAV measurement and control link are compared with this method [39, 40]. In the simulation, all methods send the same code data, in which DSSS amplification gain is 512 times. TDCS is modulated by BCSK and the sampling points are 512. The antenna number of TDCS-MIMO is 4, and the work band is in the same frequency band. The multidomain antijamming method based on CR-MIMO is used, and the sensing part can obtain more than 90% interference spectrum. All methods always have the influence of natural disturbance, and its size is represented by the SNR.

**Figure 6:**
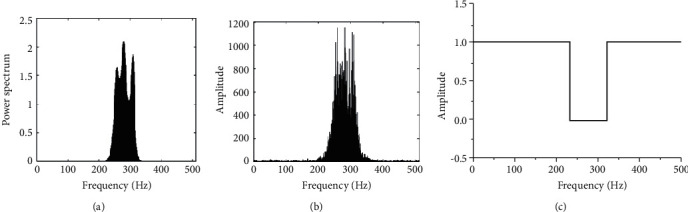
Power, frequency, and basic-function amplitude spectrum of narrowband noise jamming. (a) Power spectrum. (b) Frequency spectrum. (c) Amplitude spectrum.

If the narrowband noise is about 15% of the spectrum, as shown in Figures 6(a) and 6(b), the figure is jamming signal power spectrum (a), (b) is jamming signal based on FFT spectrum, set up a fixed threshold of 0.3. The basis function amplitude spectrum generated is as shown in Figure 6(c), covering more than 90% of the interference spectrum.

**Figure 7:**
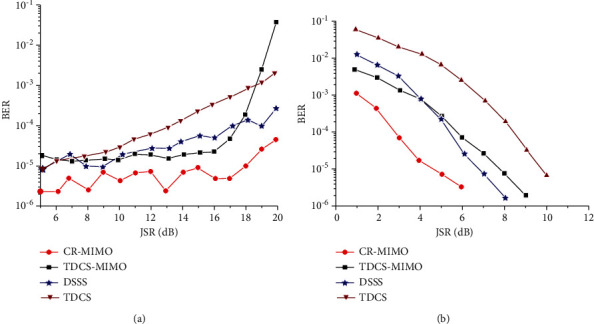
Bit error rate comparison of antijamming methods under narrowband noise jamming. (a) JSR. (b) SNR.

Figure 7(a) is the relationship between dry letter ratio and error rate under the condition of 8 dB receiving SNR.  Figure 7(b) is the relationship between SNR and error rate of 8 dB dry letter ratio.

**Figure 8:**
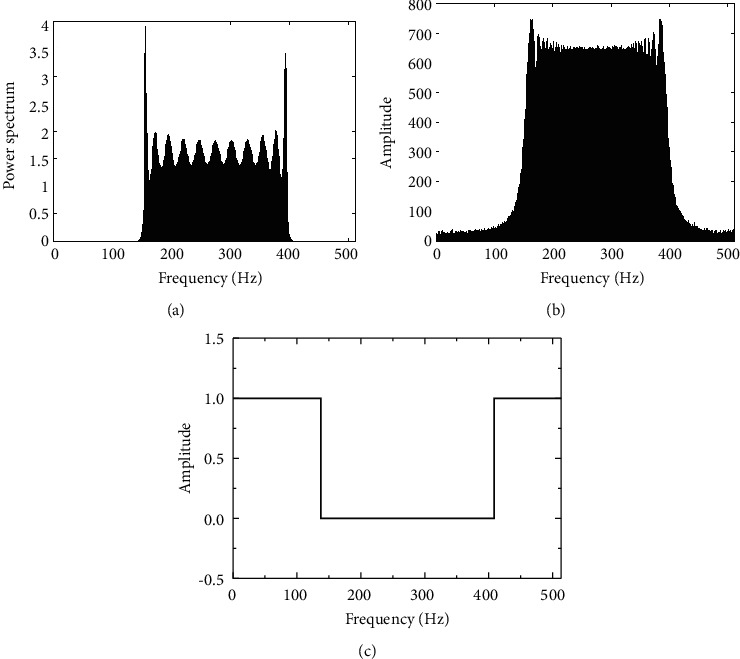
Power, frequency, and basic-function amplitude spectrum of swept frequency jamming. (a) Power spectrum. (b) Frequency spectrum. (c) Amplitude spectrum.

For the TDCS-MIMO method and the method of TDCS in similar narrow-band interference principle, the former adopts MIMO-OSTBC coding and combined ML decoding can effectively restrain the effect of natural disturbance in the channel, so the performance of TDCS-MIMO narrowband interference is better than that of TDCS method, as shown in Figure 7(a). With the increase of SNR, this advantage is obvious, as shown in Figure 7(b). DSSS is mainly used for narrowband interference, the interference is small, and its TDCS-MIMO has similar narrowband interference effect; with the augmentation of the interference, the ability of DSSS antijamming performance is even worse than that of TDCS method. Because the MIMO multidomain anti-interference methods against the narrow band interference are through perception signal transmission and jumping out of the scope of interference on the interference frequency band, it is undetected by spread spectrum interference frequency interference suppression So, compared with other methods of anti-interference, CR-MIMO multidomain antijamming method can reduce the error rate of one order of magnitude, and error performance is more stable, conforming to the theory law of BER. The narrowband interference frequency is swept by 40% scope, as shown in Figure 8.  Figure 8(a) is jamming signal power spectrum; Figure 8(b) is jamming signal based on FFT spectrum. The fixed threshold is set to 0.3; its basis function amplitude spectrum generated as shown in Figure 8(c). From the results, it can be seen, the amplitude spectrum can cover more than 90% of the interference spectrum.

**Figure 9:**
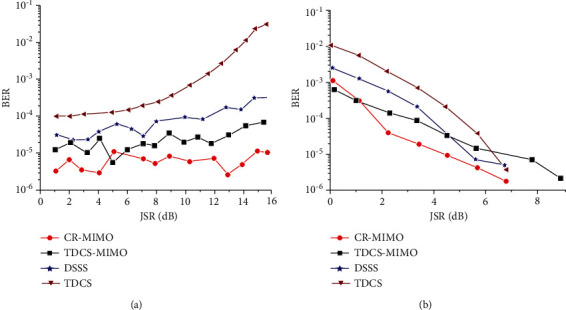
Bit error rate comparison of antijamming methods under narrowband swept frequency jamming. (a) Change of JSR. (b) Change of SNR.

Figure 9 is the relationship between dry letter ratio and error rate under the condition of 8 dB receiving SNR. It can be seen that TDCS-MIMO is still better than TDCS method for its resistance to narrow-band interference, as mentioned above. The anti-interference ability of DSSS method is poor, and the anti-interference performance deals with the interference power, and the greater the interference is, the more obvious the anti-interference performance will be, until the anti-interference is not effective. Compared with other anti-interference methods, the error code performance of the CR-MIMO multidomain anti-interference method is still relatively stable, and the anti-interference effect is better than the above three methods. The error rate can be reduced by about 0.3∼0.4 orders of magnitude.

**Figure 10:**
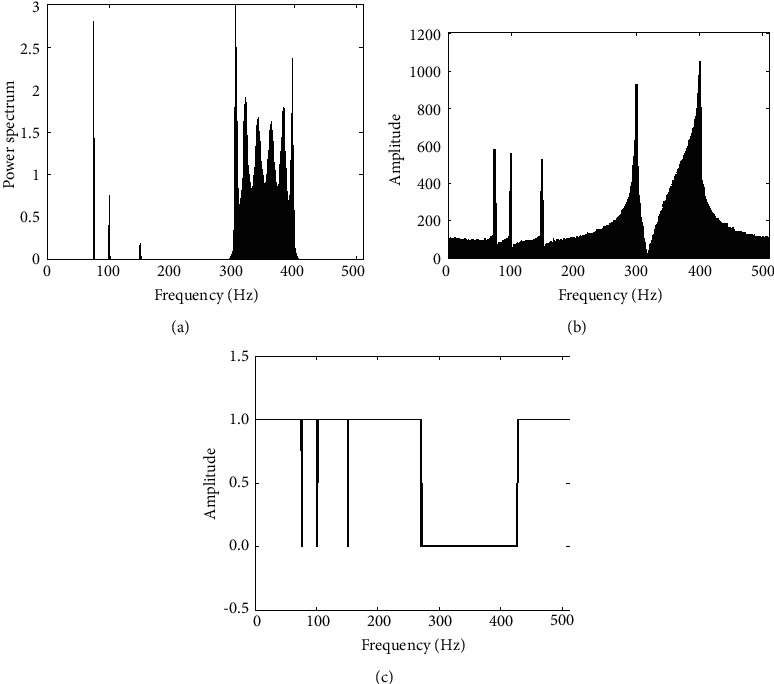
Power, frequency, and basic-function amplitude spectrum of narrowband series-wave and polyphony jamming. (a) Power spectrum. (b) Frequency spectrum. (c) Amplitude spectrum.

When more than 20% of the narrowband continuous wave is interferenced, the result is shown in Figure 10.  Figure 10(a) shows jamming signal power spectrum.  Figure 10(b) shows jamming signal based on FFT spectrum; fixed threshold is 0.3. The basis function amplitude spectrum generated is as shown in Figure 10(c), which also can cover more than 90% of the interference spectrum.

**Figure 11:**
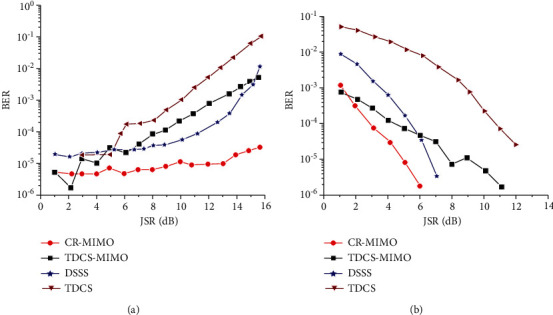
Bit error rate comparison of antijamming methods under narrowband series-wave and polyphony jamming. (a) Change of JSR. (b) Change of SNR.

Figure 11 is the relation between dry letter ratio and error rate under the condition of 8 dB receiving SNR. TDCS-MIMO is obviously better than the narrowband interference suppression method, and the method of DSSS antijamming performance is between the two; these three methods all show some of the narrowband continuous wave interference resistance, but with the augmentation of the interference, the anti-interference performance declined. And compared based on the BER performance of CR-MIMO multidomain anti-interference methods, it is stable, and anti-interference effect is better than the former three, in the dry letter than more than 10 dB, its BER can be decreased by more than about 0.5 even an order of magnitude.

**Figure 12:**
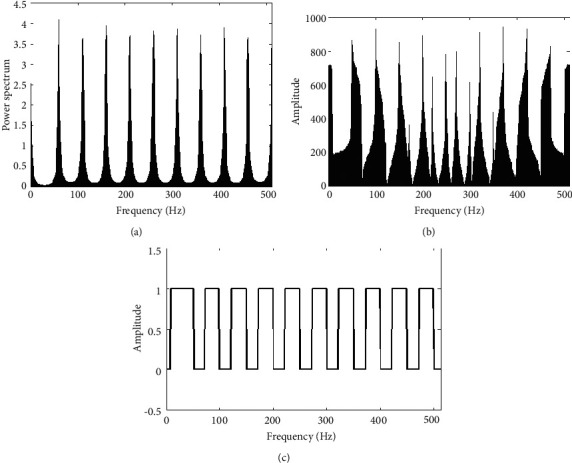
Power, frequency, and basic-function amplitude spectrum of wideband pectination jamming. (a) Power spectrum. (b) Frequency spectrum. (c) Amplitude spectrum.

When the spectrum shape of the interference signal is as the broadband comb, the result is shown in Figure 12. Figure 12(a) shows jamming signal power spectrum.  Figure 12(b) shows jamming signal based on FFT spectrum.  Figure 12(c) generates a graph for the amplitude spectrum of the basis function, assuming that the sensing part can also obtain more than 90% of the interference spectrum.

**Figure 13:**
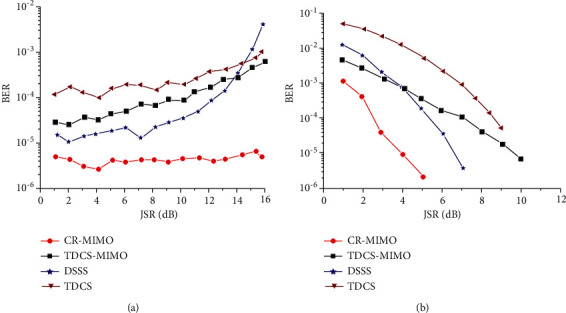
Bit error rate comparison of antijamming methods under wideband pectination jamming. (a) Change of JSR. (b) Change of SNR.

Because the multidomain anti-interference method of CR-MIMO has broadband frequency characteristics, when broadband interference does not cover all antenna frequencies, data transmission can be carried out by undisturbed antenna frequency band.  Figure 13(a) is the relationship between dry letter ratio and error rate under the condition of 10 dB receiving SNR.  Figure 13(b) is the relationship between SNR and error rate of 10 dB dry letter ratio. Because the TDCS-MIMO method and the TDCS method can be completely interfered with by the frequency band, the error rate of both is extremely high from Figures 13(a) and 13(b), which also indicates that the two methods have insufficient anti-broadband interference. The DSSS can suppress some disturbance through related operation, which can be seen from Figure 13, compared with the previous two methods, the error code performance is relatively good. And multidomain antijamming method based on CR-MIMO is the *m* sequence related to receive MIMO space-time encoding and decoding, the error rate on the basis of DSSS further reducing the one order of magnitude, and Figure 13(b) shows the relationship between SNR and BER properties meet the same theory.

**Figure 14:**
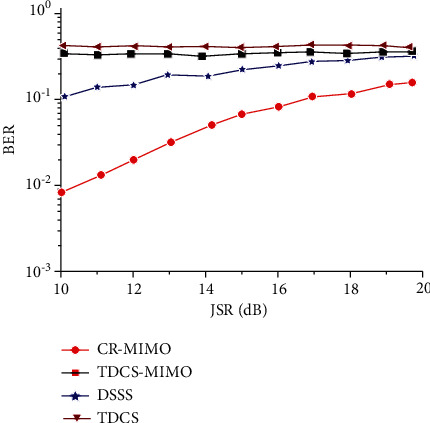
Bit error rate comparison of antijamming methods under wideband noise jamming.

When the cable is disturbed by the strong broadband noise, the simulation mainly increases the noise to flood the signal, and the relationship between the dry letter ratio and the error rate is as shown in Figure 14. It can be seen that the TDCS-MIMO method and TDCS method are difficult to find the suitable undisturbed frequency for information transmission due to the large-power noise interference. DSSS can suppress partial interference through related operation, but the anti-interference effect is still not obvious. The multidomain anti-interference method based on CR-MIMO is also affected by the interference, and the error rate is increased. However, because the MIMO diversity can improve the reliability of the system, the error performance is slightly better than the first three methods.

In conclusion, compared with the TDCS method, the multidomain anti-interference method based on MIMO is able to resist the natural interference through the space-time set and the combined ML decoding implemented by OSTBC. Compared with the method of DSSS interference attenuation, this method can achieve better anti-interference effect through using CR to find the frequency hole to achieve interference avoidance. Compared with TDCS-MIMO method, it can compensate for the interference of perceived omission by combining the spread spectrum code. In the airspace beam informs directional interference, it is difficult to guarantee full multidirectional interference suppression; this method does not limit the direction of the interference, and the receiver signal processing method is adopted to suppress the interference and has robustness against interference.

## 7. Conclusion

In this paper, the MIMO space-time coding with full-point gain is applied to the UAV measurement and control link to improve the reliability of the link. Respectively are given under the condition of nature and existence enemy drones MIMO system input and output model of data link, orthogonal space-time block coding and decoding principle, and provide theoretical basis for the subsequent anti-interference research.

Cognitive radio technology is studied based on CR multidomain anti-interference techniques of MIMO, perception, spread spectrum, and MIMO orthogonal space-time block coding together, through the coordination of space, time, frequency, and element diversity in order to realize effective for UAV data link the narrowband interference suppression, and gives the principle of the technology and the detailed mathematical model of the sending and receiving ends of anti-interference and analyzes its antijamming performance.

Through the antijamming simulation experiment of typical narrowband noise, the number of continuous wave, frequency sweep and broadband comb, broadband noise and interference suppression of the CR-MIMO multidomain, the results show that this technology can make full use of interference spectrum, realize a class-effective part of the spectrum characteristics of interference suppression, and is an effective method for interference suppression for future work.

## Data Availability

All data included in this study are available from the corresponding author upon request.
